# Socioeconomic disparities in cancer survival: Relation to stage at diagnosis, treatment, and centralization of patients to accredited hospitals, 2005–2014, Japan

**DOI:** 10.1002/cam4.5332

**Published:** 2022-10-13

**Authors:** Satomi Odani, Takahiro Tabuchi, Tomoki Nakaya, Toshitaka Morishima, Kayo Nakata, Yoshihiro Kuwabara, Mari Kajiwara Saito, Chaochen Ma, Isao Miyashiro

**Affiliations:** ^1^ Cancer Control Center Osaka International Cancer Institute Osaka Japan; ^2^ Department of Oncology Osaka University Graduate School of Medicine Suita Japan; ^3^ Graduate School of Environmental Studies Tohoku University Sendai Japan

**Keywords:** area deprivation index, cancer control, cancer survival, population‐based analysis, socioeconomic disparities

## Abstract

**Background:**

Cancer survival varies by socioeconomic status in Japan. We examined the extent to which survival disparities are explained by factors relevant to cancer control measures (promoting early‐stage detection, standardizing treatment, and centralizing patients to government‐accredited cancer hospitals [ACHs]).

**Methods:**

From the Osaka Cancer Registry, patients diagnosed with solid malignant tumors during 2005–2014 and aged 15–84 years (*N* = 376,077) were classified into quartiles using the Area Deprivation Index (ADI). Trends in inequalities were assessed for potentially associated factors: early‐stage detection, treatment modality, and utilization of ACH (for first contact/diagnosis/treatment). 3‐year all‐cause survival was computed by the ADI quartile. Multivariable Cox regression models were used to assess survival disparities and their trends through a series of adjustment for the potentially associated factors.

**Results:**

During 2005–2014, the most deprived ADI quartile had lower rates than the least deprived quartile for early‐stage detection (42.6% vs. 48.7%); receipt of surgery (58.1% vs. 64.1%); and utilization of ACH (83.5% vs. 88.4%). While rate differences decreased for receipt of surgery and utilization of ACH (Annual Percent Change = −3.2 and − 11.9, respectively) over time, it remained unchanged for early‐stage detection. During 2012–2014, the most deprived ADI quartile had lower 3‐year survival than the least deprived (59.0% vs. 69.4%) and higher mortality (Hazard Ratio [HR] = 1.32, adjusted for case‐mix): this attenuated with additional adjustment for stage at diagnosis (HR = 1.23); treatment modality (HR = 1.20); and utilization of ACH (HR = 1.19)

**Conclusions:**

Despite improvements in equalizing access to quality cancer care during 2005–2014, survival disparities remained. Interventions to reduce inequalities in early‐stage detection could ameliorate such gaps.

## 1 INTRODUCTION

Disparities in cancer survival in relation to socioeconomic status (SES) are well‐documented globally.^1^, ^2^, ^3^, ^4^, ^5^, ^6^, ^7^, ^8^, ^9^, ^10^, ^11^ As measured either at the individual or ecological levels, patients in lower SES groups (i.e., less affluent, less educated, unemployed, or underinsured) are more likely to be diagnosed with cancer at later stages^7^, ^8^, ^9^, ^11^ and less likely to receive optimal medical services,^8^, ^10^ which leads to higher mortality among these groups than their less deprived counterparts. Such disparities have been observed even in countries that assure comprehensive access to healthcare,^1^, ^2^, ^3^, ^4^, ^5^, ^6^ including Japan where its universal health coverage lets people freely choose health care facilities to attend with a nationally uniform fee schedule.^12^, ^13^

Previous Japanese studies have documented disparities in cancer survival based on municipality‐based indicators such as male unemployment rate and percentage of college graduates, or individual occupational information.^6^, ^14^, ^15^, ^16^, ^17^ Ito et al. used the area‐based deprivation index, a composite measure of neighborhood socioeconomic status, and found that absolute gaps in 5‐year survival of cancer patients had not narrowed during 1993–2004 in Japan.^5^ While these studies provide important evidence for understanding the landscape of socioeconomic disparities in Japan, they largely rely on information obtained from a limited number of individuals or information collected prior to the introduction of recent cancer control measures. Thus, there is a need for more comprehensive and recent assessment to inform public health efforts for reducing socioeconomic disparities in cancer patients.

The Health Japan 21 (second term), 10‐year national agenda for health promotion that began in 2013, has set reduction of health disparities as one of the major goals.^18^ In accordance with this, the “Basic Plan to Promote Cancer Control Program” has been in effect since 2007 to promote equalization of cancer care.^19^ Under the program, medical facilities that have the advanced capacity, experience, and leadership in cancer care have been accredited by either the Japanese national or prefectural governments (accredited cancer hospitals, ACHs hereinafter).^20^ the ACH scheme aims to facilitate specialized training for medical professionals, practicing the standard of care, and patients' proactive choice to utilize ACHs. The scheme is also expected to promote early detection of cancers by ensuring access to screening services and quality care for all populations regardless of their place of residence. As of 2020 in Osaka, the third most populated prefecture in Japan, a total of 66 (17 national and 49 prefectural) ACHs were allocated throughout the prefecture. Recent studies demonstrated that patients who underwent surgery at the national or prefectural ACHs had higher 3‐year survival than their counterparts who were treated at non‐designated hospitals,^21^ and that survival rates of patients with common solid cancers did not differ across ACHs after adjusting for case mix.^22^

While several studies support the effectiveness of the current cancer control measures in improving population‐level survival,^21^, ^22^ it remains understudied as to whether such measures could also address the existing socioeconomic disparities in cancer care and survival. In light of the above, the objectives of the present study are to (1) describe socioeconomic disparities and their trends in cancer care and survival, and (2) examine the extent to which socioeconomic disparities in cancer survival are explained by factors that are relevant to cancer control measures (i.e., promoting early‐stage detection, standardizing treatment, and centralizing patients to ACHs) in Japan. In this study, we utilized the area‐based deprivation index rather than individual characteristics as a measure of socioeconomic status, given that cancer control measures and other interventions designed to reduce health disparities are often implemented at the local level rather than targeting individuals.

## 2 MATERIALS AND METHODS

### 2.1 Data source

We obtained data from the Osaka Cancer Registry (OCR), the database of all cancer cases in Osaka prefecture (8.9 million population in 2010), Japan.^23^, ^24^ The OCR is one of the largest population‐based cancer registries in the world that meet the international qualifications of comparability, validity, timeliness, and completeness.^25^ Since the database was introduced in the early 1960s, the OCR has gathered patients' basic information such as sex, age, date of diagnosis and death, cancer site and histology,^26^ cancer stage, treatment modality, and medical facilities that patients attended for first contact, diagnosis, and first‐course treatment. Follow‐up for the vital status of the cancer patients is routinely performed using death certificates.

### 2.2 Study population

The study subjects comprised of individuals who were diagnosed with one of 15 malignant solid tumors defined by the International Classification of Diseases 10th edition (ICD‐10)^27^ (oral cavity/pharynx [C00‐14], esophagus [C15], stomach [C16], colorectum [C18‐20], liver [C22], gallbladder [C23, C24], pancreas [C25], larynx [C32], lung [C33, C34], breast [female] [C50], uterus [C53‐55], ovary [C56], prostate [C61], kidney/urinary tract/bladder [C64‐68], and thyroid [C73]) during 2005–2014 and aged 15–84 years. The analyses excluded a total of 37,162 (9.0%) individuals who were notified by death certificate only (DCO) (*N* = 26,042; 6.3%) or missing accurate residential address (*N* = 32,803; 7.9%), resulting in the analytical cohort of 376,077 individuals.

### 2.3 Variables

#### 2.3.1 Area‐based Socioeconomic status (Area Deprivation Index)

The Area Deprivation Index (ADI), a composite index of neighborhood residential characteristics of Japan was constructed by Nakaya using a comparable method to the Breadline Britain poverty measure and European transnational ecological deprivation measure.^28^, ^29^ Using official statistics from the 2010 Census, ADI was calculated as weighted sums of poverty‐related census variables (Appendix). Methodological details have been reported elsewhere.^30^ Assignment of ADI to each record of the OCR was conducted by geocoding patients' residential addresses and identifying the ADI corresponding to the respective areas at the *chocho‐aza* level (the smallest administrative unit with 3000 inhabitants on average, roughly comparable to a European parish or a U.S. block group).

#### 2.3.2 Factors relevant to cancer control measures

From a public health perspective, we assessed the following three factors that are relevant to policy, medical or behavioral interventions and have possibly affected socioeconomic disparities in cancer survival in Japan: early‐stage detection (defined as detection at a localized cancer stage), treatment modality (assessed with separate variables for receipt of surgery [open surgery, laparoscopic surgery, video‐assisted thoracic surgery, and/or endoscopic resection], radiotherapy, and/or chemotherapy as first‐course treatment), and utilization of ACH (yes/no). Cancer staging in this study was based on the summary stating system in Surveillance, Epidemiology, and End Results (SEER) program.^31^ As of March 2020, there were 66 ACHs throughout Osaka prefecture. Based on the assumption that these 66 facilities had played a leading role in centralization and standardization of cancer medical services and offered quality care since before/around the 2007 Basic Plan to Promote Cancer Control Program, we created a dichotomous variable to indicate whether patients attended any of those 66 facilities for first contact, diagnosis, and/or first‐course treatment.

#### 2.3.3 Other characteristics

Other characteristics assessed in this study included calendar year of diagnosis, sex, age (15–54; 55–64; 65–74; and 75–84 years old), and cancer site.

### 2.4 Statistical analysis

We used the ADI quartile of the Osaka general population to classify the study cohort into four groups (Q1, least deprived – Q4, most deprived). Descriptive analysis was conducted by cross‐tabulating the ADI quartile and demographic or clinical characteristics. To investigate inequalities by ADI quartile in early‐stage detection, treatment modality, and utilization of ACH, we calculated absolute indicators (rate difference and between‐group variance) and relative indicators of inequalities (rate ratio and index of disparity) within each of the calendar years during 2005–2014. Annual Percent Change (APC) was computed to examine the trends for the respective indicators with the statistical significance threshold set at *p* < 0.05. Analyses on inequality indicators were conducted using the software HD*Calc and Joinpoint, both developed by the U.S. National Cancer Institute.^32^, ^33^, ^34^, ^35^ Joinpoint regression was performed using the default setting of the software. The minimum number of observations from a joinpoint to either end of data was set to two, and the minimum number of observations between joinpoints was also set to two with the maximum number of joinpoint set to one.

And 3‐year all‐cause survival was estimated by cohort analysis for the following diagnostic periods: 2005–2007, 2008–2011, and 2012–2014. We did not examine cause‐specific or relative survival because information on cause of death was not collected in the OCR and reference mortality data by ADI quartile were not available. Hazard ratios (HRs) were computed by Cox proportional hazard models adjusted for calendar year, sex, age, and cancer site to compare 3‐year all‐cause mortality by ADI quartile. To test trends in survival disparities during 2005–2014, a variable representing the interaction between ADI quartile and calendar year was included in Cox proportional hazard models. The direction of trend was determined according to the interaction coefficient (>1 as widening: <1 as narrowing). Statistical significance of the interaction term was assessed using likelihood ratio tests (*p* < 0.05). We calculated Cramer's V to check for correlations among the independent variables and confirmed that none of the assessed variables were strongly correlated (i.e. >0.5). Cox‐Snell residual test was used to assess the goodness of fit. The Proportional hazards assumption was checked by using statistical tests and graphical diagnostics based on the scaled Schoenfeld residuals and supported by a non‐significant relationship between residuals and time.

A series of Cox proportional hazard models were used to examine the extent to which disparities in survival could be explained by potentially associated factors (early‐stage detection, treatment modality, and utilization of ACH). The base model (Model 1) estimated the HRs by ADI quartile adjusted for calendar year, sex, age, and cancer site. Additional adjustments were made sequentially for the stage at diagnosis (Model 2), treatment modality (Model 3), and utilization of ACH (Model 4). We also fitted separate models to decompose the explanatory effect of each of the three potentially associated factors. Survival analyses were conducted using the survival package of R version 4.0.3.

This study was approved by the Institutional Review Board of Osaka International Cancer Institute (approval number: 19143). We obtained the dataset with no personally identifiable information from the OCR and independently processed it in accordance with the Act on Promotion of Cancer Registries. The OCR data are not publicly accessible and are available only on request due to privacy and ethical restrictions.

## 3 RESULTS

Table 1 shows characteristics of patients by ADI quartile. During 2005–2014, the numbers of patients in Q1 (least deprived), Q2, Q3, and Q4 (most deprived) were 81,193 (21.6%), 95,834 (25.5%), 100,518 (26.7%), and 98,532 (26.2%), respectively. Among patients in the least deprived ADI quartile, 48.7% were diagnosed at an early (localized) stage. This proportion was lower in more deprived ADI quartiles: 46.1% (Q2), 44.2% (Q3), and 42.6% (Q4), respectively. And 64.1% of patients in the least deprived ADI quartile underwent surgery while a lower proportion of patients received surgery in more deprived ADI quartiles: 62.0% (Q2), 60.6% (Q3), and 58.1% (Q4), respectively. The proportion that utilized ACHs for first contact, diagnosis, and/or treatment was 88.4% in the least deprived ADI quartile, which decreased with elevating levels of deprivation to 87.8% (Q2), 85.8% (Q3), and 83.5% (Q4).

**TABLE 1:** Characteristic of patients by ADI quartile, 2005–2014, Osaka Cancer Registry, Japan

	ADI Q1 (least deprived)	ADI Q2	ADI^a^ Q3	ADI^a^ Q4 (most deprived)	Overall
	(*N* = 81,193, 21.6% of all patients)	(*N* = 95,834, 25.5% of all patients)	(*N* = 100,518, 26.7% of all patients)	(*N* = 98,532, 26.2% of all patients)	(*N* = 376,077)
Characteristics	*N* (%)	*N* (%)	*N* (%)	*N* (%)	*N* (%)
Sex					
Male	47,989 (59.1%)	57,270 (59.8%)	60,431 (60.1%)	59,967 (60.9%)	225,657 (60.0%)
Female	33,204 (40.9%)	38,564 (40.2%)	40,087 (39.9%)	38,565 (39.1%)	150,420 (40.0%)
Age (years)					
15–54	12,561 (15.5%)	12,324 (12.9%)	12,133 (12.1%)	10,103 (10.3%)	47,121 (12.5%)
55–64	19,256 (23.7%)	21,400 (22.3%)	21,732 (21.6%)	20,453 (20.8%)	82,841 (22.0%)
65–74	27,901 (34.4%)	34,751 (36.3%)	37,568 (37.4%)	38,413 (39.0%)	138,633 (36.9%)
75–84	21,475 (26.4%)	27,359 (28.5%)	29,085 (28.9%)	29,563 (30.0%)	107,482 (28.6%)
Cancer site					
Oral cavity/pharynx	2001 (2.5%)	2535 (2.6%)	2657 (2.6%)	2654 (2.7%)	9847 (2.6%)
Esophagus	2704 (3.3%)	3219 (3.4%)	3410 (3.4%)	3582 (3.6%)	12,915 (3.4%)
Stomach	13,675 (16.8%)	16,734 (17.5%)	17,627 (17.5%)	16,877 (17.1%)	64,913 (17.3%)
Colorectum	13,339 (16.4%)	15,992 (16.7%)	17,293 (17.2%)	16,961 (17.2%)	63,585 (16.9%)
Liver	4926 (6.1%)	6774 (7.1%)	7501 (7.5%)	8481 (8.6%)	27,682 (7.4%)
Gallbladder	1659 (2.0%)	2120 (2.2%)	2334 (2.3%)	2293 (2.3%)	8406 (2.2%)
Pancreas	3318 (4.1%)	3884 (4.1%)	4115 (4.1%)	3919 (4.0%)	15,236 (4.1%)
Larynx	623 (0.8%)	711 (0.7%)	813 (0.8%)	906 (0.9%)	3053 (0.8%)
Lung	10,576 (13.0%)	13,866 (14.5%)	15,214 (15.1%)	15,965 (16.2%)	55,621 (14.8%)
Breast (female)	9495 (11.7%)	9926 (10.4%)	9701 (9.7%)	8635 (8.8%)	37,757 (10.0%)
Uterus	3040 (3.7%)	3355 (3.5%)	3291 (3.3%)	3075 (3.1%)	12,761 (3.4%)
Ovary	1219 (1.5%)	1269 (1.3%)	1286 (1.3%)	1161 (1.2%)	4935 (1.3%)
Prostate	8317 (10.2%)	8305 (8.7%)	7842 (7.8%)	6971 (7.1%)	31,435 (8.4%)
Kidney/urinary tract/bladder	4751 (5.9%)	5466 (5.7%)	5747 (5.7%)	5598 (5.7%)	21,562 (5.7%)
Thyroid	1550 (1.9%)	1678 (1.8%)	1687 (1.7%)	1454 (1.5%)	6369 (1.7%)
Stage at diagnosis					
Localized	39,530 (48.7%)	44,173 (46.1%)	44,438 (44.2%)	41,937 (42.6%)	170,078 (45.2%)
Regional	20,456 (25.2%)	25,250 (26.3%)	27,064 (26.9%)	26,506 (26.9%)	99,276 (26.4%)
Distant	13,497 (16.6%)	17,693 (18.5%)	19,935 (19.8%)	20,168 (20.5%)	71,293 (19.0%)
Unknown	7710 (9.5%)	8718 (9.1%)	9081 (9.0%)	9921 (10.1%)	35,430 (9.4%)
Surgery					
Yes	52,075 (64.1%)	59,426 (62.0%)	60,941 (60.6%)	57,289 (58.1%)	229,731 (61.1%)
No	23,331 (28.7%)	30,022 (31.3%)	32,749 (32.6%)	33,835 (34.3%)	119,937 (31.9%)
Unknown	5787 (7.1%)	6386 (6.7%)	6828 (6.8%)	7408 (7.5%)	26,409 (7.0%)
Radiotherapy					
Yes	10,184 (12.5%)	11,541 (12.0%)	12,005 (11.9%)	11,629 (11.8%)	45,359 (12.1%)
No	64,342 (79.2%)	76,887 (80.2%)	80,428 (80.0%)	78,123 (79.3%)	299,780 (79.7%)
Unknown	6667 (8.2%)	7406 (7.7%)	8085 (8.0%)	8780 (8.9%)	30,938 (8.2%)
Chemotherapy					
Yes	25,493 (31.4%)	31,163 (32.5%)	33,209 (33.0%)	32,233 (32.7%)	122,098 (32.5%)
No	49,282 (60.7%)	57,568 (60.1%)	59,578 (59.3%)	58,000 (58.9%)	224,428 (59.7%)
Unknown	6418 (7.9%)	7103 (7.4%)	7731 (7.7%)	8299 (8.4%)	29,551 (7.9%)
Utilization of ACHs^b^					
Yes	71,743 (88.4%)	84,159 (87.8%)	86,285 (85.8%)	82,235 (83.5%)	324,422 (86.3%)
No	9450 (11.6%)	11,675 (12.2%)	14,233 (14.2%)	16,297 (16.5%)	51,655 (13.7%)

Abbreviations: ACH, designated cancer care hospital; ADI, area deprivation index.

^a^ ADI was calculated as a composite index of neighborhood characteristics using the 2010 Census data and classified into four groups using quartiles of Osaka general population (Q1 least deprived—Q4 most deprived).

^b^ ACHs are accredited medical care facilities by either the Japanese national or prefectural governments for their advanced capacity, experience, and leadership in cancer care. A dichotomous variable was created to indicate whether patients attended any of the 66 ACHs in Osaka prefecture for first contact, diagnosis, and/or first‐course treatment.

Table 2 and Table S1 demonstrate 3‐year all‐cause survival by ADI quartile. Overall survival rate increased from 56.1% during 2005–2007, 60.9% during 2008–2011, to 63.7% during 2012–2014. During 2012–2014, 3‐year survival in each ADI quartile was 69.4% (Q1), 64.8% (Q2), 62.6% (Q3), and 59.0% (Q4). In each period and most of the population subgroups, there was a consistent gradient with lower survival in more deprived ADI quartiles. Among overall, while the excess mortality risk (HR adjusted for calendar year, sex, age, and cancer site) in the most deprived ADI quartile was 1.28 (95% CI = 1.25–1.32, vs. least deprived) during 2005–2007 and 1.32 (95% CI = 1.28–1.35, vs. last deprived) during 2012–2014, we did not observe a statistically significant trend throughout 2005–2014 (Table S1). Stratified by sex, age, and cancer site, disparities in survival significantly widened during 2005–2014 among males, those aged 65–74 years, those diagnosed with esophageal cancer and lung cancer (all *p* < 0.05). Disparities significantly narrowed among individuals diagnosed with liver cancer and laryngeal cancer (both *p* < 0.05).

**TABLE 2:** 3‐year all‐cause survival by ADI quartile, 2005–2014, Osaka Cancer Registry, Japan

Characteristics	2005–2007					2008–2011					2012–2014				
	ADI^a^ Q1	ADI^a^ Q2	ADI^a^ Q3	ADI^a^ Q4	Overall	ADI^a^ Q1	ADI^a^ Q2	ADI^a^ Q3	ADI^a^ Q4	Overall	ADI^a^ Q1	ADI^a^ Q2	ADI^a^ Q3	ADI^a^ Q4	Overall
	(*N* = 20,242, 21.8% of all patients)	(*N* = 24,224, 25.7% of all patients)	(*N* = 26,028, 26.7% of all patients)	(*N* = 25,663, 25.8% of all patients)	(*N* = 96,157)	(*N* = 32,303, 21.7% of all patients)	(*N* = 37,868, 25.5% of all patients)	(*N* = 39,519, 26.6% of all patients)	(*N* = 39,064, 26.3% of all patients)	(*N* = 148,754)	(*N* = 28,648, 21.8% of all patients)	(*N* = 33,742, 25.7% of all patients)	(*N* = 34,971, 26.7% of all patients)	(*N* = 33,805, 25.8% of all patients)	(*N* = 131,166)
Overall	62.3	57.3	54.5	51.6	56.1	66.8	62.7	59.1	55.9	60.9	69.4	64.8	62.6	59.0	63.7
Sex															
Male	56.8	51.8	49.0	45.8	50.4	62.6	57.9	53.9	51.2	56.0	65.7	60.4	58.3	54.3	59.4
Female	70.0	65.4	62.8	60.6	64.4	73.0	69.9	67.0	63.4	68.2	74.7	71.3	69.2	66.4	70.3
Age (years)															
15–54	80.0	73.4	71.4	69.6	73.8	82.4	79.5	78.5	74.8	79.0	85.0	81.7	80.8	77.3	81.5
55–64	67.1	63.4	60.5	56.7	61.7	72.5	68.4	65.6	61.4	66.9	74.2	70.6	67.8	65.4	69.5
65–74	61.1	57.0	53.9	51.3	55.3	67.4	63.4	58.8	57.3	61.3	70.4	66.0	63.1	60.9	64.8
75–84	46.4	42.9	41.4	39.3	42.2	52.0	49.7	46.5	43.8	47.7	56.1	53.0	52.1	48.4	52.1
Cancer site															
Oral cavity/pharynx	63.8	58.7	58.7	55.8	58.9	66.4	61.4	60.0	57.6	61.0	69.5	64.0	60.4	55.6	62.0
Esophagus	36.5	34.3	33.3	30.7	33.4	45.8	45.1	37.4	37.6	41.0	54.0	48.7	44.6	40.7	46.7
Stomach	59.9	56.4	53.7	51.1	54.9	65.9	61.7	58.5	57.2	60.6	69.9	64.5	62.2	60.4	64.0
Colorectum	71.2	67.0	65.9	63.6	66.7	73.6	70.4	69.5	67.6	70.1	76.4	73.2	70.1	68.4	71.7
Liver	44.5	42.7	39.6	36.0	40.1	47.2	46.7	43.9	40.9	44.2	47.6	43.6	45.2	43.3	44.7
Gallbladder	23.2	21.5	21.5	19.3	21.2	29.9	25.9	25.0	22.1	25.4	32.8	29.3	30.4	24.8	29.1
Pancreas	11.4	10.0	9.2	7.9	9.5	13.0	13.3	11.3	9.8	11.8	17.7	14.7	14.9	11.3	14.6
Larynx	81.1	75.1	75.1	66.7	73.7	79.9	75.8	77.9	75.3	77.1	77.8	77.9	73.5	76.9	76.4
Lung	36.1	30.7	29.5	27.8	30.5	41.1	35.9	32.5	29.1	34.0	45.6	39.8	36.8	34.6	38.7
Breast (female)	91.8	90.4	89.2	89.8	90.3	93.0	91.8	91.2	90.0	91.6	91.5	91.1	91.1	90.4	91.0
Uterus	79.2	77.6	75.1	74.4	76.6	81.3	81.8	79.2	75.0	79.3	82.5	82.4	80.7	77.4	80.9
Ovary	63.4	59.4	58.5	56.0	59.3	66.3	66.0	62.6	55.2	62.5	71.3	67.3	66.1	62.4	66.9
Prostate	87.8	86.4	82.5	83.5	85.1	91.4	89.5	86.0	84.4	88.1	91.3	90.0	88.4	86.0	89.0
Kidney/urinary tract/bladder	72.7	68.7	65.5	62.1	67.1	73.9	71.2	66.8	64.1	68.7	72.2	72.2	69.4	63.4	69.2
Thyroid	89.7	91.5	90.3	83.3	88.7	95.0	90.9	91.4	88.4	91.5	92.5	89.9	89.8	86.0	89.7
Stage at diagnosis															
Localized	86.8	84.6	82.0	79.6	83.2	88.9	86.5	84.4	81.9	85.4	89.6	87.0	86.0	83.4	86.5
Regional	58.4	53.7	52.2	50.1	53.3	63.2	59.6	57.1	53.8	58.1	67.5	63.3	61.9	58.8	62.6
Distant	15.7	13.2	12.9	13.0	13.5	18.3	16.2	14.7	14.0	15.6	20.1	18.3	17.8	15.6	17.7
Unknown	47.1	41.5	39.1	36.2	40.5	51.3	44.8	40.3	37.5	43.2	32.4	29.3	24.4	21.7	26.5
Surgery^b^															
Yes	77.8	75.1	72.7	71.1	74.0	80.9	78.0	75.9	73.9	77.1	82.8	80.2	78.5	76.6	79.5
No	33.1	28.6	26.5	25.7	28.0	39.6	35.7	31.9	29.4	33.6	43.2	37.5	36.2	32.5	36.9
Unknown	44.5	40.2	37.2	33.1	38.2	53.7	47.6	43.2	38.7	45.4	44.5	37.8	31.1	29.4	35.4
Radiotherapy															
Yes	60.6	54.5	50.9	49.3	53.4	68.0	63.6	57.4	54.9	60.8	71.7	66.3	64.5	59.7	64.9
No	64.0	59.0	56.2	53.2	57.8	67.5	63.4	60.4	57.2	61.9	70.5	64.6	64.0	60.6	65.1
Unknown	54.9	51.3	49.1	46.6	50.1	56.9	51.1	45.7	42.5	48.7	45.0	38.3	32.2	29.9	36.0
Chemotherapy															
Yes	46.5	41.5	41.1	39.3	41.8	51.2	47.5	45.3	43.9	46.7	56.0	51.5	50.5	47.4	51.1
No	71.7	66.9	63.1	59.8	65.1	76.1	72.2	68.0	64.1	69.8	78.2	73.8	71.7	67.5	72.6
Unknown	56.4	53.0	50.2	47.7	51.4	57.7	51.8	46.6	43.0	49.4	45.0	38.4	32.3	30.2	36.2
Utilization of ACHs^c^															
Yes	64.8	60.2	58.0	56.0	59.5	53.0	48.3	43.8	40.3	45.3	71.1	66.7	64.9	61.7	65.9
No	44.2	38.9	36.8	33.7	37.4	68.5	64.5	61.4	58.9	63.1	57.1	51.5	48.7	44.1	49.7

Abbreviations: ACH, designated cancer care hospital; ADI, area deprivation index; CI, confidence interval.

^a^ ADI was calculated as a composite index of neighborhood characteristics using the 2010 Census data and classified into four groups using quartiles of Osaka general population (Q1 least deprived—Q4 most deprived).

^b^ Surgeries included open surgery, laparoscopic surgery, video‐assisted thoracic surgery, and endoscopic resection.

^c^ ACHs are accredited medical care facilities by either the Japanese national or prefectural governments for their advanced capacity, experience, and leadership in cancer care. A dichotomous variable was created to indicate whether patients attended any of the 66 ACHs in Osaka prefecture for first contact, diagnosis, and/or first‐course treatment.

Table 3 presents 3‐year all‐cause mortality HRs by ADI quartile and their changes through a series of adjustments. During 2012–2014, when adjusted for calendar year, sex, age, and cancer site (Model 1), HRs were higher in more deprived ADI quartiles (HR = 1.13 [95% CI = 1.10–1.16], 1.20 [95% CI = 1.17–1.23], and 1.32 [1.28–1.35] in Q2, Q3, and Q4, respectively) compared to the least deprived ADI quartile. After additional adjustment for cancer stage at diagnosis (Model 2), the HRs dropped for all ADI quartiles, and this was more apparent in the most deprived ADI quartile (HR = 1.10 [95% CI = 1.07–1.13], 1.14 [95% CI = 1.11–1.18], and 1.23 [1.20–1.26] in Q2, Q3 and Q4, respectively). Further adjustment for treatment modality (Model 3) reduced the HRs to 1.08 (95% CI = 1.05–1.11), 1.13 (95% CI = 1.10–1.16), and 1.20 (95% CI = 1.17–1.23) in Q2, Q3 and Q4, respectively. Final adjustment for utilization of ACH (Model 4) slightly reduced the HR for Q4 to 1.19 (95% CI = 1.16–1.22).

**TABLE 3:** Step‐by‐step adjustment of 3‐year all‐cause mortality hazard ratio and trend in disparity by ADI quartile, 2005–2014, Osaka Cancer Registry, Japan

	Model 1		Model 2		Model 3		Model 4	
	Adjusted for: Calendar year, sex, age at diagnosis, and cancer site		Adjusted for: Model 1 + Stage at diagnosis		Adjusted for: Model 2 + Treatment modality (surgery,^b^ radiotherapy, chemotherapy)		Adjusted for: Model 3 + Utilization of ACHs^c^	
Calendar year, ADI^a^	HR	95% CI	HR	95% CI	HR	95% CI	HR	95% CI
2005–2007								
Q1	ref.		ref.		ref.		ref.	
Q2	1.12	1.09–1.16	1.10	1.07–1.14	1.08	1.04–1.11	1.07	1.03–1.10
Q3	1.19	1.16–1.23	1.15	1.12–1.18	1.13	1.10–1.16	1.10	1.07–1.13
Q4	1.28	1.25–1.32	1.22	1.19–1.26	1.19	1.16–1.23	1.14	1.11–1.17
2012–2014								
Q1	ref.		ref.		ref.		ref.	
Q2	1.13	1.10–1.16	1.10	1.07–1.13	1.08	1.05–1.11	1.08	1.04–1.11
Q3	1.20	1.17–1.23	1.14	1.11–1.18	1.13	1.10–1.16	1.13	1.10–1.16
Q4	1.32	1.28–1.35	1.23	1.20–1.26	1.20	1.17–1.23	1.19	1.16–1.22
2005–2014: Trend in disparity								
		—		—		—		Widened

*Note*: 3‐year all‐cause mortality HRs were calculated for the two time periods (2005–2007 & 2012–2014) to depict the state of disparities in the first and last 3 years of the study period; however, data from all the 10 calendar years (2005–2014) were encompassed in the trend analyses. The trend in disparity was tested with Cox proportional hazard models using the interaction term between ADI quartile and calendar year (*p* < 0.05).

Abbreviations: ACH, designated cancer care hospitals; ADI, area deprivation index; CI, confidence interval; HR, hazard ratio; ref., referent; −, trend not statistically significant.

^a^ ADI was calculated as a composite index of neighborhood characteristics using the 2010 Census data and classified into four groups using quartiles of Osaka general population (Q1 least deprived – Q4 most deprived).

^b^ Surgeries included open surgery, laparoscopic surgery, video‐assisted thoracic surgery, and endoscopic resection.

^c^ ACHs are accredited medical care facilities by either the Japanese national or prefectural governments for their advanced capacity, experience, and leadership in cancer care. A dichotomous variable was created to indicate whether patients attended any of the 66 ACHs in Osaka prefecture for first contact, diagnosis, and/or first‐course treatment.

Similarly, during 2005–2007, HRs in Q2, Q3, and Q4 was reduced through a series of adjustment. Notably, more apparent reduction was observed for 2005–2007 compared to that for 2012–2014 when the model was adjusted for utilization of ACH; from Model 3 to Model 4, HRs decreased from 1.08 (95% CI = 1.04–1.11) to 1.07 (95% CI = 1.03–1.10) in Q2: 1.13 (95% CI = 1.10–1.16) to 1.10 (95% CI = 1.07–1.13) in Q3: and 1.19 (95% CI = 1.16–1.23) to 1.14 (95% CI = 1.11–1.17) in Q4. Looking at the trends in survival disparity, a significant increase was observed during 2005–2014 when the model was adjusted for all the potentially associated factors assessed (Model 4). Decomposed effects of each of the factors are presented in Table S2.

Table 4 shows annual rates and rate differences of early‐stage detection, treatment modality, and utilization of ACH by the ADI quartile. During 2005–2014, the rate that underwent surgery was constantly highest in the least deprived ADI quartile and lowest in the most deprived ADI quartile: the rate difference significantly decreased from 7.0 percentage points (2005) to 6.1 percentage point (points (APC = −3.2)). Similarly, for utilization of ACH, the rate difference significantly decreased from 9.5 percentage points (2005) to 2.5 percentage points (2014) (APC = −11.9). To ascertain these trends, we also computed between‐group variance, rate ratio, and index of disparity (Table S3). For receipt of surgery, the rate ratio significantly decreased from 1.14 (2005) to 1.10 (2014) (APC = −0.5). For utilization of ACH, all the assessed inequality indicators significantly decreased during 2005–2014 (all *p* < 0.05). For early‐stage detection, significant trends were not observed for any of the four inequality indicators assessed.

**TABLE 4:** Rates and rate differences of early‐stage detection, treatment modality, and utilization of ACHs by ADI quartile, 2005–2014, Osaka Cancer Registry, Japan

	Rate (%)				Absolute inequality		
Calendar year	ADI^a^ Q1	ADI^a^ Q2	ADI^a^ Q3	ADI^a^ Q4	Rate difference	Annual percent change	p‐trend
Early‐stage detection							
2005	42.1	39.2	37.5	34.7	7.4		
2006	45.1	41.4	39.3	38.0	7.1		
2007	44.3	42.6	41.0	39.1	5.2		
2008	46.0	43.5	42.3	39.6	6.5		
2009	46.3	44.8	43.6	42.3	4.1		
2010	49.3	46.8	43.9	42.5	6.8		
2011	49.2	47.7	44.7	44.1	5.1		
2012	52.2	49.7	48.4	46.9	5.3		
2013	52.8	50.1	47.6	46.7	6.1		
2014	54.1	50.2	49.3	47.1	7.0	−1.0	0.665
Surgery^b^							
2005	59.0	55.6	54.4	51.9	7.0		
2006	62.5	59.0	58.6	55.7	6.8		
2007	64.6	61.9	60.6	56.7	7.8		
2008	63.3	62.1	60.1	57.3	6.0		
2009	63.5	61.5	59.8	58.9	4.7		
2010	63.8	62.8	59.6	57.7	6.2		
2011	64.4	63.1	61.4	59.4	4.9		
2012	66.1	64.4	63.5	61.2	5.0		
2013	65.1	63.1	63.0	59.6	5.5		
2014	66.5	63.9	62.7	60.4	6.1	−3.2	0.048
Radiotherapy							
2005	11.0	10.5	11.5	11.1	1.0		
2006	11.5	11.1	11.7	12.0	0.9		
2007	11.7	11.7	11.6	11.4	0.3		
2008	12.7	11.8	11.5	11.3	1.4		
2009	12.9	12.8	11.4	11.5	1.5		
2010	12.7	12.2	11.7	11.7	1.0		
2011	13.1	12.0	12.3	11.9	1.2		
2012	13.1	12.7	12.4	11.7	1.4		
2013	12.9	12.6	12.4	12.5	0.5		
2014	13.0	12.3	12.5	12.5	0.7	−0.8	0.896
Chemotherapy							
2005	27.6	29.2	29.4	29.2	1.8		
2006	29.0	30.2	31.0	30.4	2.1		
2007	29.6	30.9	31.5	32.0	2.4		
2008	32.0	32.9	32.8	33.2	1.2		
2009	32.8	34.4	34.5	34.0	1.6		
2010	31.8	33.2	33.1	33.6	1.8		
2011	31.9	33.4	34.1	33.0	2.2		
2012	33.5	34.7	35.7	34.5	2.2		
2013	32.1	32.5	33.9	32.9	1.9		
2014	31.8	32.3	32.9	33.1	1.3	−1.4	0.588
Utilization of ACHs^c^							
2005	84.9	84.0	80.0	75.4	9.5		
2006	88.2	86.8	84.0	81.8	6.4		
2007	89.9	88.5	87.0	83.7	6.2		
2008	88.6	88.0	86.6	83.2	5.4		
2009	88.6	88.4	86.3	83.3	5.3		
2010	89.7	89.6	86.5	84.2	5.4		
2011	89.8	89.3	87.6	86.2	3.7		
2012	89.4	88.9	87.5	86.1	3.3		
2013	87.4	87.2	85.5	84.1	3.3		
2014	86.7	86.8	86.0	84.3	2.5	−11.9	<0.001

*Note*: Annual Percent Changes and *p*‐values for trends were calculated using Joinpoint regression models.

Abbreviations: ADI, area deprivation index; ACH, designated cancer care hospitals.

^a^ ADI was calculated as a composite index of neighborhood characteristics using the 2010 Census data and classified into four groups using quartiles of Osaka general population (Q1 least deprived – Q4 most deprived).

^b^ Surgeries included open surgery, laparoscopic surgery, video‐assisted thoracic surgery, and endoscopic resection.

^c^ ACHs are accredited medical care facilities by either the Japanese national or prefectural governments for their advanced capacity, experience, and leadership in cancer care. A dichotomous variable was created to indicate whether patients attended any of the 66 ACHs in Osaka prefecture for first contact, diagnosis, and/or first‐course treatment.

## 4 DISCUSSION

This is the first study to investigate socioeconomic disparities in cancer care and survival in Japan in relation to factors that can be addressed through cancer control measures. During 2005–2014, there was a socioeconomic gradient in early‐stage detection, receipt of surgery, and utilization of ACH with lower rates in more deprived ADI quartiles. While absolute inequalities decreased for receipt of surgery and utilization of ACH over the 10 years, it remained unchanged for early‐stage detection. During 2012–2014, the most deprived ADI quartile had a 10.4 percentage points lower 3‐year survival than the least deprived ADI quartile (59.0% vs. 69.4%) which corresponded with a 32% higher mortality hazard (HR = 1.32) after adjusting for case‐mix. This differential decreased to HR = 1.19 when additional adjustment was made for the stage at diagnosis, treatment modality, and utilization of ACH, suggesting that stage at diagnosis was the most influential explanatory factor. While these findings demonstrate progress toward equalization of access to quality cancer care, survival disparities did not narrow over the 10 years. Furthermore, the need to strengthen certain domains of cancer control, such as the reduction of inequalities in early‐stage detection, is highlighted.

During 2005–2014, a majority of patients utilized ACHs in all ADI quartiles, and inequalities in utilization of ACH attenuated. The greatest increase in the ACH utilization rate was seen in the most deprived ADI quartile from 75.4% in 2005 to 84.3% in 2014. This indicates that the current centralization scheme may have been especially effective to connect individuals living in deprived neighborhoods to ACHs who would otherwise not attend those facilities. Furthermore, in the series of adjustment, disparities in cancer survival during 2012–2014 scarcely changed when the model was adjusted for utilization of ACH, while an apparent reduction was seen in the same analysis for 2005–2007. This is likely due to the progress that had been made during 2005–2014 in equalizing access to ACHs which might have achieved a near‐optimal level by 2014.

Decreased inequality was also seen for receipt of surgery, but not for radiotherapy or chemotherapy. While radiotherapy and chemotherapy can be used for either curative or palliative purposes, surgery plays a vital role in radical treatment for solid tumors.^36^, ^37^, ^38^ Possible explanations for the decreased inequality in receipt of surgery include enhanced standardization of treatment modality and centralization of patients to more surgically skilled hospitals.^21^, ^39^ However, in assessing equalization of cancer treatment, it should be noted that treatment modality is decided considering clinical suitability of individual patients such as stage at diagnosis, presence of comorbidities, and overall physical functional ability to tolerate invasive effects of the treatment. These factors may mediate the association between individuals' SES and the type of treatment received.^6^, ^39^

Consistent with findings from the previous studies,^6^, ^11^ our step‐by‐step adjustment analysis demonstrated that the stage at diagnosis was the most influential factor to describe disparities in cancer survival. We also revealed that inequality in early‐stage detection did not narrow during 2005–2014 despite that early‐stage detection rates constantly increased in all ADI quartiles. In Japan, although there have been efforts to promote early‐stage detection through free‐of‐charge or affordable cancer‐screening programs at the subnational levels, low uptake of cancer screening has been a major public health concern.^40^ While the national guideline recommends annual or biennial screening for five common cancers (stomach, colorectum, lung, breast, and cervical cancers), just above 40% of all individuals in the recommended age group reported receiving a screening test in 2019.^41^ A potential reason for this is the absence of a national, comprehensive program to administer cancer screening which currently relies on coordinated efforts of local municipalities, workplaces, and individuals who voluntarily uptake screening.^42^ This might have led to inequalities in early‐stage detection according to one's place of residence and employment status. At the individual level, lack of knowledge and insufficient financial resources are major barriers to cancer screening and follow‐up after an abnormal screening result.^8^, ^11^, ^43^, ^44^ Taken together with these facts, our findings suggest the need for individual‐ or community‐based interventions to further enhance early‐stage detection, especially in deprived populations.

It is important to note that, even after controlling for the stage at diagnosis and the other associated factors, disparities in cancer survival remained statistically significant, and there was a widening trend during 2005–2014. Possible explanations for the remaining disparities are as follows. First, patients' survival used as an end‐point reflects all‐cause death which might have resulted in overestimation of disparities in cancer survival. It is well‐documented that individuals in lower SES have a heavier burden of morbidities and shorter life expectancy than those in higher SES.^45^, ^46^ Further investigation is needed to account for these underlying disparities; comprehensive data that contains cause‐specific survival and reference mortality data linked to ADI information are desired. Second, inequalities in lifestyle factors (e.g. smoking, drinking, diet, and exercise habit) and compliance with treatment may also predict disparities in prognosis.^45^, ^46^, ^47^ As uptake of cancer screening, the above‐mentioned factors depend on individual knowledge, awareness, and social networks.^6^ Monitoring these factors can help understand the trends and causes of survival disparities that have not been well‐addressed under the current cancer control scheme.

Our study is subject to several limitations. First, we used ADI, area‐based ecological information of residents' SES. Individuals may have been misclassified as the inferences at the area level do not directly transfer to individuals. However, given that cancer control measures are often implemented at the local or regional levels, it is reasonable to use an area‐based ecological indicator as a tool for assessing cancer disparities. Second, the HRs assessed by Cox proportional hazard models should be interpreted with caution as we were unable to adjust for all potential confounders (e.g., psychosocial, medical, or behavioral factors) that are not collected by the OCR. Third, we excluded invalid registrations (i.e., DCO cases and those lacking accurate residential address) from the analysis which may have resulted in biased cohort characteristics. However, the DCO rate of our study cohort was 6.3%, which met the international standard for completeness (DCO <10%),^48^ and only a small proportion (2.7%) was further excluded due to lack of accurate residential addresses. Thus, we assume that such bias was kept minimum in our analyses. Fourth, cancer staging in this study was based on the SEER summary staging system, as the OCR lacks TNM‐staging information. Therefore, for some cancer sites, localized cancers in this study could include stage II cancers based on TNM criteria which may have resulted in misclassification of early stage detection. Finally, the generalizability of the findings may be limited due to the geodemographic uniqueness of Osaka that has a comparatively high density of population and ACHs. Despite these limitations, we have demonstrated the progress that has been made and the potential that socioeconomic disparities in cancer survival can be reduced by further public health efforts. This should be taken into consideration by public health officials and policymakers to help design, implement, and evaluate future interventions to achieve health equity.

## 5 CONCLUSION

During 2005–2014, there was a socioeconomic gradient in 3‐year survival of cancer patients with lower survival probabilities in more deprived ADI quartiles, while inequalities in receipt of surgery and utilization of ACH attenuated over the 10 years. The most deprived ADI quartile had 1.32 times higher 3‐year mortality hazard during 2012–2014 compared to the least deprived ADI quartile. This disparity was reduced to 1.19 times when adjusted for early stage detection, treatment modality, and utilization of ACH, suggesting that stage at diagnosis was the most influential factor to describe survival disparities. Continued implementation of a structural approach to intervening in the medical care provision system, in combination with the individual‐ or community‐based interventions to narrow inequalities in early‐stage detection, could help reduce survival disparities among cancer patients.

## AUTHOR CONTRIBUTIONS

**Takahiro Tabuchi:** Conceptualization (equal); data curation (equal); methodology (equal); supervision (equal); validation (equal); visualization (equal); writing – review and editing (equal). **Tomoki Nakaya:** Conceptualization (equal); data curation (equal); formal analysis (equal); methodology (equal); validation (equal); writing – review and editing (equal). **Toshitaka Morishima:** Data curation (equal); investigation (equal); project administration (equal); resources (equal); validation (equal); writing – review and editing (equal). **Kayo Nakata:** Data curation (equal); investigation (equal); project administration (equal); resources (equal); validation (equal); writing – review and editing (equal). **Yoshihiro Kuwabara:** Data curation (equal); investigation (equal); project administration (equal); resources (equal); validation (equal); writing – review and editing (equal). **Mari Kajiwara Saito:** Data curation (equal); investigation (equal); project administration (equal); resources (equal); validation (equal); writing – review and editing (equal). **Chaochen Ma:** Data curation (equal); investigation (equal); resources (equal); validation (equal); writing – review and editing (equal). **Isao Miyashiro:** Data curation (equal); investigation (equal); project administration (equal); resources (equal); validation (equal); writing – review and editing (equal).

## FUNDING INFORMATION

This work was supported by JSPS KAKENHI Grant (JP22K10548) and Health, Labor and Welfare Sciences Research Grant (H30‐Gantaisaku‐Ippan‐009).

## CONFLICT OF INTEREST

The authors have no conflicts of interest to disclose.

## ETHICS APPROVAL STATEMENT

This study was approved by the Institutional Review Board of Osaka International Cancer Institute (approval number: 19143).

## Supporting information


Appendix S1:

## Data Availability

The Osaka Cancer Registry data are not publicly accessible and are available only on request due to privacy and ethical restrictions.
