# Supplementary material for: Socioeconomic disparities in cancer survival: Relation to stage at diagnosis, treatment, and centralization of patients to accredited hospitals, 2005–2014, Japan
Source: Cancer Med. 2022 Oct 13;12(5):6077–91. doi: 10.1002/cam4.5332 (PMC10028172; doi:10.1002/cam4.5332)
Supplement: Supplementary file 1 — Appendix S1: [file CAM4-12-6077-s001.docx]

**Supplementary Table 1.** The disparity in 3-year all-cause survival and its trend, 2005-2014, Osaka Cancer Registry, Japan

|  | 2005-2007 | | | | |  | 2012-2014 | | | | |  | 2005-2014 |
| --- | --- | --- | --- | --- | --- | --- | --- | --- | --- | --- | --- | --- | --- |
|  | 3-year survival probability | | |  | Excess risk |  | 3-year survival probability | | |  | Excess risk |  |  |
| Characteristics | ADI^1^ Q1 | ADI^1^ Q4 | Probability difference  (Q1-Q4) |  | HR (95%CI) (Adjusted for calendar year, sex, age, and cancer site accordingly) (Q4) |  | ADI^1^ Q1 | ADI^1^ Q4 | Probability difference (Q1-Q4) |  | HR (95%CI) (Adjusted for calendar year, sex, age, and cancer site accordingly) (Q4) |  | Trend in disparity (Adjusted for calendar year, sex, age, and cancer site accordingly) |
| Overall | 62.3 (61.6-62.9) | 56.1 (55.8-56.4) | 10.7 |  | 1.28 (1.25-1.32) |  | 69.4 (68.9-69.9) | 59.0 (58.5-59.5) | 10.4 |  | 1.32 (1.28-1.35) |  | - |
| Sex |  |  |  |  |  |  |  |  |  |  |  |  |  |
| Male | 56.8 (55.9-57.7) | 50.4 (50.0-50.8) | 11.0 |  | 1.31 (1.26-1.35) |  | 65.7 (65.0-66.5) | 54.3 (53.6-55.0) | 11.4 |  | 1.36 (1.32-1.41) |  | Widened |
| Female | 70.0 (69.0-71.0) | 64.4 (64.0-64.9) | 9.4 |  | 1.23 (1.17-1.30) |  | 74.7 (73.9-75.5) | 66.4 (65.6-67.2) | 8.3 |  | 1.22 (1.16-1.28) |  | - |
| Age (years) |  |  |  |  |  |  |  |  |  |  |  |  |  |
| 15-54 | 80.0 (78.6-81.3) | 73.8 (73.0-74.6) | 10.4 |  | 1.52 (1.38-1.68) |  | 85.0 (84.0-86.1) | 77.3 (75.9-78.8) | 7.7 |  | 1.45 (1.30-1.61) |  | - |
| 55-64 | 67.1 (65.9-68.4) | 61.7 (61.1-62.3) | 10.4 |  | 1.33 (1.25-1.41) |  | 74.2 (73.1-75.3) | 65.4 (64.2-66.6) | 8.8 |  | 1.33 (1.25-1.43) |  | - |
| 65-74 | 61.1 (59.9-62.3) | 55.3 (54.8-55.8) | 9.8 |  | 1.29 (1.23-1.35) |  | 70.4 (69.5-71.3) | 60.9 (60.0-61.7) | 9.5 |  | 1.37 (1.31-1.43) |  | Widened |
| 75-84 | 46.4 (45.1-47.9) | 42.2 (41.6-42.9) | 7.1 |  | 1.18 (1.13-1.24) |  | 56.1 (55.0-57.2) | 48.4 (47.5-49.3) | 7.7 |  | 1.23 (1.18-1.29) |  | - |
| Cancer site |  |  |  |  |  |  |  |  |  |  |  |  |  |
| Oral cavity/pharynx | 63.8 (59.8-68.1) | 58.9 (57.1-60.9) | 8.0 |  | 1.27 (1.06-1.52) |  | 69.5 (66.2-73.0) | 55.6 (52.4-59.0) | 13.9 |  | 1.50 (1.27-1.78) |  | - |
| Esophagus | 36.5 (33.1-40.3) | 33.4 (31.9-35.1) | 5.8 |  | 1.26 (1.11-1.42) |  | 54.0 (51.0-57.2) | 40.7 (38.0-43.6) | 13.3 |  | 1.48 (1.32-1.67) |  | Widened |
| Stomach | 59.9 (58.2-61.5) | 54.9 (54.2-55.7) | 8.8 |  | 1.30 (1.22-1.39) |  | 69.9 (68.6-71.2) | 60.4 (59.1-61.6) | 9.5 |  | 1.38 (1.29-1.47) |  | - |
| Colorectum | 71.2 (69.7-72.7) | 66.7 (66.0-67.5) | 7.6 |  | 1.32 (1.22-1.43) |  | 76.4 (75.2-77.6) | 68.4 (67.2-69.6) | 8.0 |  | 1.37 (1.27-1.48) |  | - |
| Liver | 44.5 (42.2-47.1) | 40.1 (39.1-41.2) | 8.5 |  | 1.26 (1.16-1.37) |  | 47.6 (45.1-50.3) | 43.3 (41.4-45.3) | 4.3 |  | 1.12 (1.03-1.23) |  | Narrowed |
| Gallbladder | 23.2 (19.6-27.5) | 21.2 (19.6-23.0) | 3.9 |  | 1.23 (1.07-1.41) |  | 32.8 (29.1-36.9) | 24.8 (21.9-28.1) | 8.0 |  | 1.21 (1.06-1.38) |  | - |
| Pancreas | 11.4 (9.4-13.8) | 9.5 (8.6-10.5) | 3.5 |  | 1.20 (1.09-1.32) |  | 17.7 (15.6-20.0) | 11.3 (9.7-13.2) | 6.4 |  | 1.22 (1.12-1.33) |  | - |
| Larynx | 81.1 (75.4-87.2) | 73.7 (70.8-76.8) | 14.4 |  | 2.01 (1.34-3.02) |  | 77.8 (72.1-84.0) | 76.9 (72.1-82.0) | 0.9 |  | 1.01 (0.68-1.49) |  | Narrowed |
| Lung | 36.1 (34.3-38.0) | 30.5 (29.7-31.2) | 8.3 |  | 1.25 (1.18-1.33) |  | 45.6 (44.1-47.3) | 34.6 (33.3-35.9) | 11.0 |  | 1.33 (1.26-1.40) |  | Widened |
| Breast (female) | 91.8 (90.7-92.9) | 90.3 (89.7-90.9) | 2.0 |  | 1.15 (0.95-1.40) |  | 91.5 (90.6-92.5) | 90.4 (89.3-91.4) | 1.1 |  | 1.05 (0.89-1.23) |  | - |
| Uterus | 79.2 (76.3-82.1) | 76.6 (75.1-78.0) | 4.8 |  | 1.13 (0.91-1.39) |  | 82.5 (80.3-84.8) | 77.4 (74.9-80.0) | 5.1 |  | 1.24 (1.02-1.50) |  | - |
| Ovary | 63.4 (58.3-68.9) | 59.3 (56.7-61.9) | 7.4 |  | 1.23 (0.96-1.57) |  | 71.3 (67.0-75.8) | 62.4 (57.5-67.6) | 8.9 |  | 1.27 (0.99-1.63) |  | - |
| Prostate | 87.8 (86.2-89.5) | 85.1 (84.2-86.1) | 4.3 |  | 1.38 (1.13-1.68) |  | 91.3 (90.3-92.2) | 86.0 (84.7-87.3) | 5.3 |  | 1.51 (1.30-1.76) |  | - |
| Kidney/urinary tract/bladder | 72.7 (70.2-75.2) | 67.1 (65.8-68.4) | 10.6 |  | 1.51 (1.31-1.73) |  | 72.2 (70.1-74.4) | 63.4 (61.3-65.6) | 8.8 |  | 1.31 (1.16-1.47) |  | - |
| Thyroid | 89.7 (86.5-93.0) | 88.7 (87.1-90.4) | 6.4 |  | 1.10 (0.72-1.67) |  | 92.5 (90.4-94.8) | 86.0 (83.0-89.1) | 6.5 |  | 1.60 (1.09-2.36) |  | - |

**Abbreviations:** ADI=Area Deprivation Index; ACH=designated cancer care hospitals; HR=hazard ratio; CI=confidence interval; -=trend not statistically significant

**Note:** All-cause survival and mortality HRs were calculated for the two time periods (2005-2007 & 2012-2014) to depict the state of disparities in the first and last 3 years of the study period; however, data from all the 10 calendar years (2005-2014) were encompassed in the trend analyses. Trend in disparity was tested with Cox proportional hazard models using the interaction term between ADI quartile and calendar year (p<0.05).

1. ADI was calculated as a composite index of neighborhood characteristics using the 2010 Census data and classified into four groups using quartiles of Osaka general population (Q1 least deprived – Q4 most deprived)

**Supplementary Table 2.** 3-year all-cause mortality hazard ratio by ADI quartile (individual adjustment for stage at diagnosis, treatment modality, and utilization of ACH), 2005-2007 & 2012-2014, Osaka Cancer Registry, Japan

|  | Adjusted for:  Calendar year, sex, age at diagnosis, cancer site | |  | Adjusted for:  Calendar year, sex, age at diagnosis, cancer site  +  Stage at diagnosis | |  | Adjusted for:  Calendar year, sex, age at diagnosis, cancer site  +  Treatment modality (surgery^2^, radiotherapy, and/or chemotherapy) | |  | Adjusted for:  Calendar year, sex, age at diagnosis, cancer site  +  Utilization of ACHs^3^ | |
| --- | --- | --- | --- | --- | --- | --- | --- | --- | --- | --- | --- |
| Calendar year, ADI^1^ | HR | 95% CI |  | HR | 95% CI |  | HR | 95% CI |  | HR | 95% CI |
| 2005-2007 |  |  |  |  |  |  |  |  |  |  |  |
| Q1 | ref. |  |  | ref. |  |  | ref. |  |  | ref. |  |
| Q2 | 1.12 | 1.09-1.16 |  | 1.10 | 1.07-1.14 |  | 1.09 | 1.05-1.12 |  | 1.11 | 1.08-1.14 |
| Q3 | 1.19 | 1.16-1.23 |  | 1.15 | 1.12-1.18 |  | 1.15 | 1.12-1.19 |  | 1.16 | 1.13-1.19 |
| Q4 | 1.28 | 1.25-1.32 |  | 1.22 | 1.19-1.26 |  | 1.22 | 1.19-1.26 |  | 1.22 | 1.19-1.26 |
| 2012-2014 |  |  |  |  |  |  |  |  |  |  |  |
| Q1 | ref. |  |  | ref. |  |  | ref. |  |  | ref. |  |
| Q2 | 1.13 | 1.10-1.16 |  | 1.10 | 1.07-1.13 |  | 1.11 | 1.08-1.14 |  | 1.13 | 1.10-1.16 |
| Q3 | 1.20 | 1.17-1.23 |  | 1.14 | 1.11-1.17 |  | 1.16 | 1.13-1.19 |  | 1.19 | 1.16-1.23 |
| Q4 | 1.32 | 1.28-1.35 |  | 1.23 | 1.20-1.26 |  | 1.25 | 1.21-1.28 |  | 1.30 | 1.26-1.33 |

**Abbreviations:** ADI=Area Deprivation Index; ACH=designated cancer care hospitals; HR=hazard ratio; CI=confidence interval; ref.=referent

1. ADI was calculated as a composite index of neighborhood characteristics using the 2010 Census data and classified into four groups using quartiles of Osaka general population (Q1 least deprived – Q4 most deprived)
2. Surgeries included open surgery, laparoscopic surgery, video-assisted thoracic surgery, and endoscopic resection
3. ACHs are accredited medical care facilities by either the Japanese national or prefectural governments for their advanced capacity, experience, and leadership in cancer care. A dichotomous variable was created to indicate whether patients attended any of the 66 ACHs in Osaka prefecture for first contact, diagnosis, and/or first course treatment

**Supplementary Table 3.** Absolute and relative inequalities and annual changes in early-stage detection, treatment modality, and utilization of ACHs by ADI quartile, 2007-2012, Osaka Cancer Registry, Japan

|  | Calendar year | | | | | | | | | | |  | |  | |  |
| --- | --- | --- | --- | --- | --- | --- | --- | --- | --- | --- | --- | --- | --- | --- | --- | --- |
| Inequality indicator | 2005 | 2006 | 2007 | 2008 | 2009 | 2010 | 2011 | 2012 | 2013 | 2014 |  | | Annual Percent Change | | p-trend | |
| Early-stage detection |  |  |  |  |  |  |  |  |  |  |  | |  | |  | |
| Rate difference | 7.35 | 7.07 | 5.19 | 6.48 | 4.08 | 6.77 | 5.08 | 5.32 | 6.12 | 6.95 |  | | -1.0 | | 0.665 | |
| Between-group variance | 7.13 | 7.20 | 3.68 | 5.43 | 2.27 | 6.83 | 4.41 | 3.81 | 5.67 | 6.30 |  | | -1.3 | | 0.771 | |
| Rate Ratio | 1.21 | 1.19 | 1.13 | 1.16 | 1.10 | 1.16 | 1.12 | 1.11 | 1.13 | 1.15 |  | | -0.6 | | 0.075 | |
| Index of disparity | 13.98 | 10.27 | 9.10 | 11.14 | 6.21 | 9.71 | 6.90 | 6.87 | 7.41 | 8.70 |  | | -5.3 | | 0.052 | |
| Surgery^1^ |  |  |  |  |  |  |  |  |  |  |  | |  | |  | |
| Rate difference | 7.05 | 6.77 | 7.85 | 6.03 | 4.67 | 6.15 | 4.93 | 4.96 | 5.48 | 6.13 |  | | -3.2 | | 0.048 | |
| Between-group variance | 6.46 | 5.77 | 7.99 | 5.22 | 3.17 | 6.04 | 3.41 | 3.18 | 3.89 | 4.90 |  | | -6.2 | | 0.071 | |
| Rate Ratio | 1.14 | 1.12 | 1.14 | 1.11 | 1.08 | 1.11 | 1.08 | 1.08 | 1.09 | 1.10 |  | | -0.5 | | 0.023 | |
| Index of disparity | 8.40 | 7.72 | 9.85 | 7.97 | 4.64 | 7.59 | 5.90 | 5.69 | 6.95 | 6.63 |  | | -3.6 | | 0.134 | |
| Radiation |  |  |  |  |  |  |  |  |  |  |  | |  | |  | |
| Rate difference | 1.00 | 0.87 | 0.33 | 1.37 | 1.53 | 0.99 | 1.19 | 1.38 | 0.49 | 0.67 |  | | -0.8 | | 0.896 | |
| Between-group variance | 0.13 | 0.10 | 0.02 | 0.27 | 0.49 | 0.17 | 0.22 | 0.25 | 0.03 | 0.07 |  | | -1.2 | | 0.919 | |
| Rate Ratio | 1.10 | 1.08 | 1.03 | 1.12 | 1.13 | 1.09 | 1.10 | 1.12 | 1.04 | 1.06 |  | | -0.2 | | 0.671 | |
| Index of disparity | 6.73 | 5.41 | 2.61 | 6.18 | 9.27 | 4.18 | 4.71 | 8.78 | 2.09 | 2.68 |  | | -6.2 | | 0.290 | |
| Chemotherapy |  |  |  |  |  |  |  |  |  |  |  | |  | |  | |
| Rate difference | 1.81 | 2.06 | 2.40 | 1.22 | 1.65 | 1.81 | 2.19 | 2.18 | 1.86 | 1.30 |  | | -1.4 | | 0.588 | |
| Between-group variance | 0.56 | 0.56 | 0.81 | 0.20 | 0.43 | 0.47 | 0.63 | 0.60 | 0.47 | 0.26 |  | | -3.8 | | 0.439 | |
| Rate Ratio | 1.07 | 1.07 | 1.08 | 1.04 | 1.05 | 1.06 | 1.07 | 1.07 | 1.06 | 1.04 |  | | -0.2 | | 0.254 | |
| Index of disparity | 6.27 | 5.52 | 6.25 | 3.05 | 4.46 | 4.77 | 4.95 | 4.25 | 3.27 | 3.09 |  | | -6.1 | | 0.023 | |
| Utilization of ACHs^2^ |  |  |  |  |  |  |  |  |  |  |  | |  | |  | |
| Rate difference | 9.52 | 6.40 | 6.21 | 5.36 | 5.33 | 5.45 | 3.69 | 3.33 | 3.28 | 2.49 |  | | -11.9 | | < 0.001 | |
| Between-group variance | 14.13 | 6.16 | 5.35 | 4.32 | 4.62 | 5.22 | 2.11 | 1.69 | 1.78 | 1.02 |  | | -21.6 | | < 0.001 | |
| Rate Ratio | 1.13 | 1.08 | 1.07 | 1.06 | 1.06 | 1.07 | 1.04 | 1.04 | 1.04 | 1.03 |  | | -0.8 | | < 0.001 | |
| Index of disparity | 10.04 | 5.58 | 5.74 | 5.42 | 5.42 | 5.17 | 3.23 | 2.90 | 3.07 | 2.63 |  | | -12.1 | | < 0.001 | |

**Abbreviations:** ADI=Area Deprivation Index; ACH=designated cancer care hospitals.

**Note:** ADI was calculated as a composite index of neighborhood characteristics using the 2010 Census data and classified into quartiles (Q1 least deprived – Q4 most deprived). Between-group difference was assessed as an indicator of absolute inequality that reflects the magnitude of deviations from the population average: index of disparity was assessed as an indicator of relative inequality that summarizes the relative distance that the population is away from the egalitarian state. Annual Percent Changes and p-values for trends were calculated using Joinpoint regression models.

1. Surgeries included open surgery, laparoscopic surgery, video-assisted thoracic surgery, and endoscopic resection
2. ACHs are accredited medical care facilities by either the Japanese national or prefectural governments for their advanced capacity, experience, and leadership in cancer care. A dichotomous variable was created to indicate whether patients attended any of the 66 ACHs in Osaka prefecture for first contact, diagnosis, and/or first-course treatment

**Appendix.** Formula to calculate Area Deprivation Index using the Japanese Census data

The score of areal deprivation index (ADI) used in this study was defined as the weighted sum of the census variables using the following formula:

ADI_i_ = k × (2.99 × proportion of old couple households_i_ + 7.57 × proportion of old single households_i_ + 17.4 × proportion of single-mother households_i_ + 2.22 × proportion of rent houses_i_ + 4.03 × proportion of sales and service workers_i_ + 6.05 × proportion of agricultural workers_i_ + 5.38 × proportion of blue caller workers_i_ + 18.3 × unemployment rate_i_),

where *i* is an area index (Japanese Chocho-Aza, roughly equivalent to a European parish or a U.S. block group) and k refers to a positive constant. The value of k was originally assigned by making a synthetic estimate of the number of ‘poverty’ households in an area.
